# Fuzzy-Logic-Based Recommendation System for Processing in Condition Monitoring

**DOI:** 10.3390/s22103695

**Published:** 2022-05-12

**Authors:** Jakub Gorski, Mateusz Heesch, Michal Dziendzikowski, Ziemowit Dworakowski

**Affiliations:** 1 Department of Robotics and Mechatronics, AGH University of Science and Technology, al. A. Mickiewicza 30, 30-059 Krakow, Poland; jgorski@agh.edu.pl (J.G.); heesch@agh.edu.pl (M.H.); 2 Airworthiness Division, Air Force Institute of Technology, ul. Ks. Boleslawa 6, 01-494 Warsaw, Poland; michal.dziendzikowski@itwl.pl

**Keywords:** PBSHM, recommendation system, condition monitoring, fuzzy logic, gearbox, fault detection

## Abstract

The development of a machine’s condition monitoring system is often a challenging task. This process requires the collection of a sufficiently large dataset on signals from machine operation, context information related to the operation conditions, and the diagnosis experience. The two referred problems are today relatively easy to solve. The hardest to describe is the diagnosis experience because it is based on imprecise and non-numerical information. However, it is essential to process acquired data to develop a robust monitoring system. This article presents a framework for a system dedicated to recommending processing algorithms for condition monitoring. It includes a database and fuzzy-logic-based modules composed within the system. Based on the contextual knowledge provided by the user, the procedure suggests processing algorithms. This paper presents the evaluation of the proposed agent on two different parallel gearboxes. The results of the system are processing algorithms with assigned model types. The obtained results show that the algorithms recommended by the system achieve a higher accuracy than those selected arbitrarily. The results obtained allow for an average of 5 to 14.5% higher accuracy.

## 1. Introduction

**Figure 1:**
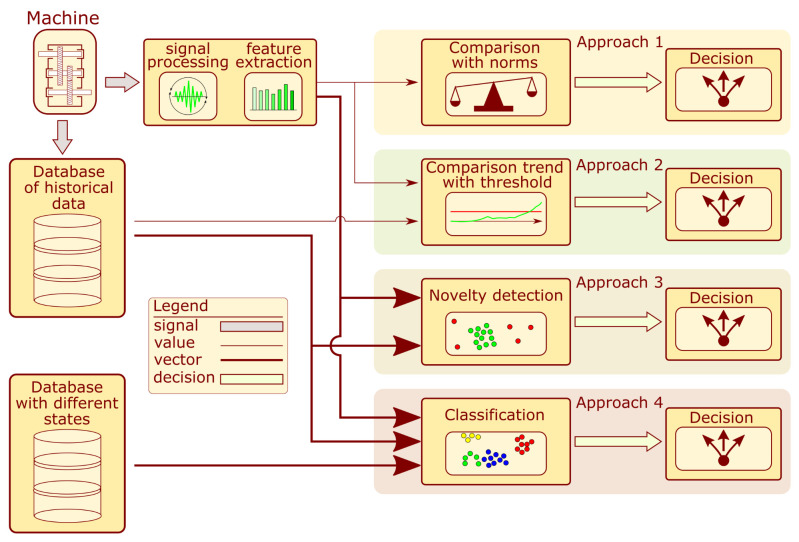
Block diagram of condition assessment system development.

Choosing an appropriate structure state assessment algorithm is a fundamental problem in condition monitoring (CM). The block diagram presenting different approaches to algorithm development is presented in Figure 1. The foundation of each system is usually the analysis of vibration signals recorded from the machine. Based on them and an in-depth analysis of the diagnostics problem for similar structures, the processing algorithms are determined to extract features that allow for a proper assessment of the structure state [1].

In approach 1, the indicator value is compared with the norms for the intact machine. If they are exceeded, an alarm is reported. These systems seem simple to develop. However, the proper establishment of a relevant standard can be challenging task. The authors of [2] describe a method for automatic threshold selection based on data distribution estimation. When historical diagnostic information is available, it is possible to develop more sophisticated systems based on a trend analysis of the calculated indicators. By determining the indicator distribution over a certain monitoring period, one can obtain a threshold value. If the value exceeds the threshold, the system triggers an alarm. Additionally, the trend analysis uses the trend slope and noise values, which are also considered when reporting alarm situations. The successfully implemented examples of this were introduced for wind [3,4] and gas [5] turbines. Unfortunately, proposing a single indicator that contains enough information is a problematic and sometimes even impossible task. Therefore, a group of features is used to address the structure assessment multidimensional problem. Instead of a threshold value, a subspace is employed to determine failure presence. If the coordinates of the new sample are outside the designated space, an alarm is reported. This approach is described in the literature as novelty detection or anomaly detection. The cited examples of this strategy are related to bridge tendons [6], where the authors developed a convolutional autoencoder. Another example dedicated to bridges is presented in [7], where the authors propose a one-class kNN model based on Mahalanobis-squared distance. Another example is described in [8], where several online novelty detection algorithms for gearboxes were proposed. When appropriate labels are assigned to multidimensional data, it is possible to develop a classifier. It extends the capabilities of novelty algorithms by dividing the feature space into subspaces that different machine states define. Some examples of this strategy are concerned with gearbox monitoring. The authors of [9] present a system based on a morphological filter and a binary tree support vector machine (BT-SVM). The second reviewed work [10] related to gearboxes describes the application of decision trees and SVM. Other reviewed works were dedicated to the monitoring of bearings. In [11,12], the authors proposed a quadratic classifier; in [13], features were extracted using an autoencoder and applied for state assessment. Other approaches are depicted in [14,15], where the authors describe classifiers trained on the dataset with a low number of labels. In [14], the researchers used a convolutional autoencoder, and in [15], a semi-supervised deep learning model was developed.

The approaches described above are templates. Detailed implementation requires specialist knowledge and experience in developing diagnostic systems for similar devices. Therefore, an idea emerged of proposing a recommendation system that would support the work of diagnostic system designers. The proposed system should tackle two main challenges. Firstly, each structure requires a specific monitoring approach due to potential sources of failure. Thus, the developed system should determine the analyzed structure profile based on contextual information. The second issue is related to a measure of the recommendation quality. In CM, such information is achievable as diagnostic results. However, it is often qualitative in form, which is not suitable as feedback information in recommendation system development. Therefore, it was necessary to propose a quantitative measure appropriate for the system.

Considering the paragraph, it is reasonable to apply fuzzy logic systems (FLSs). This would allow for the incorporation of expert knowledge into the recommendation system. The only example of an autonomous recommendation system for SHM can be found in a paper by Kung et al. [16]. It is an ontology-based approach that contains a glossary of terms developed for data exchange between collaborating teams. At each step, the system interacts with the user via mediators. This part of the system searches a constructed ontology to propose tools useful at a given analysis step.

The data exchange that underpins the proposed decision support system is also related to a new monitoring approach called population-based structural health monitoring (PBSHM). In contrast to the current monitoring strategy, where only data collected for a structure are used for the condition assessment, in PBSHM the information on the similar object population is applied. If the objects are identical and operate under very similar conditions, then it is possible to transfer diagnostic information to determine the range of damage indices considered to be the undamaged condition of the object [17]. For structures that do not satisfy the above requirement, the similarity measure must be calculated [18] in order to determine which part of the diagnostic information can be transferred [19,20]. The recommendation system presented in this paper is a solution to the problem of knowledge transfer between non-identical structures. It utilizes a database containing data for different objects operating under various conditions. The system proposes the processing algorithms that provided the best result when monitoring structures with the highest similarity to the analyzed one.

Thus, the main goal of this paper was to propose a framework for a system dedicated to supporting the process of processing algorithms development for condition monitoring. The system requires a database with historical data from previously analyzed structures and contextual information described in a quantified manner. Based on these, the system suggests processing algorithms that are the most appropriate for the considered monitoring problem. The remaining part of the paper is organized as follows: Section 2 presents a short overview of recommendation systems and the usage of fuzzy logic in recommendation systems. Section 3 contains details of the proposed fuzzy-logic-based processing recommendation system. Section 4 describes the framework implementation dedicated to gearboxes. The developed system evaluation and results are explained in Section 5. Finally, Section 6 presents the conclusions.

## 2. Related Work

Recommendation systems are inseparably related to the Internet and especially web browsers. Without them, it would be impossible to search the massive collections of data stored. The foundation of all recommendation agents is information filtration [21]. Four basic approaches are mentioned among researchers dealing with this topic: collaborative-filtering-based, demographic-filtering-based, content-based, and knowledge-based approaches [22]. In addition to these, other concepts have also been developed, such as social-network-based, context-awareness-based, and grouped-recommendation approaches, as well as hybrid methods that are a combination of those previously mentioned [23].

In a content-based approach, the system uses item features to recommend another item similar to user preferences based on their previous actions or explicit feedback [24]. The collaborative filtering approach employs the recommendation pipeline similarity of users and items to provide suitable items. The collaborative filtering models can recommend an item to user A based on the interests of a similar user, B [24]. Demographic filtering has in common with collaborative filtering the similarity between users. However, the similarity measure is calculated based on demographic data such as age, gender, and occupation. Users are classified into stereotyped sets. The system generates recommendations that have found the highest approval in this group [22].

The recommendation system is knowledge-based when it makes recommendations based on specific user queries. It may encourage the user to provide a series of rules or guidance on what results are required or provide an example of an item [25]. There are two types of knowledge-based recommendation approaches: case-based and constraint-based approaches. Both utilize similar fractures of knowledge, but they differ in the way solutions are calculated. Specifically, case-based recommenders generate recommendations based on similarity metrics, whereas constraint-based recommenders mainly exploit predefined knowledge bases that contain explicit rules about how to relate user requirements with item features [22]. Defining the problem as described allows for the application of FLS. Such a system contains implemented rules which establish the main operation and generalize in unforeseen cases. The mentioned approach enables the development of recommendation systems that rely on imprecise information provided by users.

The FLS was implemented in various recommendation applications [26]. The most commonly used application is the recommendation of services or wares. One such system is described in [27] for consumer advice in computer parts selection. Another customer application is presented in the paper [28], where, based on contextual information such as time duration and desired condition, the system selects the best offer for the buyer.

Fuzzy logic systems have found utilization in other applications not related to e-commerce. The majority of them are not dedicated to engineering problems. One can be found in [29], which supports the online learning process, proposing suggestions about class activities. As feedback, the students’ engagement is measured by the facial expressions captured by the camera. Another application described in paper [30] is a system recommending a diet for people with diabetes. The authors proposed a system structure based on an ontology model representing domain knowledge as a graph of concepts. Another based on an ontology system [31] is built on a patient’s medical history and can recommend diet and medication. Medical measurements such as blood pressure, sugar level, or heart rate are used as input data. Along with the Internet of Things network, it provides the doctor with data by enabling constant monitoring of the patient’s condition. The last presented in this review of examples of fuzzy-logic-based recommenders are presented in [32]. An application of a developed knowledge-based system is for a field engineers’ work assistant by suggesting the most suitable tasks.

The overview presented in the above paragraphs revealed that the dominant trend in recommendation systems using fuzzy logic is the knowledge-based structure. Therefore in the developed recommendation system, the knowledge-based approach is also employed. According to the system’s terminology, the cases are previously monitored machines, and the constraints are context information that includes experiment conditions, the variability of measured signals, and expected diagnostic problems. The following section is devoted to the description of the framework.

## 3. System Framework

**Figure 2:**
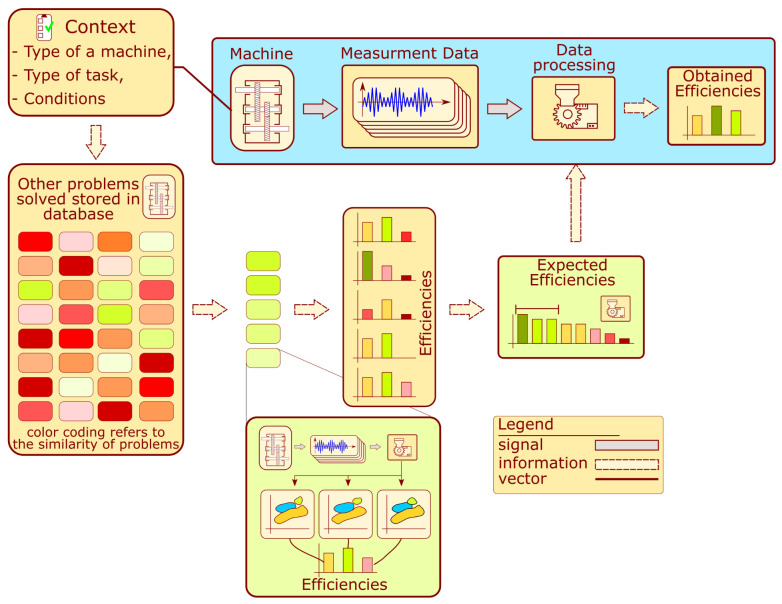
Principle of processing recommendation algorithm.

The operation of the system is described by the block diagram shown in Figure 2. The monitoring system development for a given machine requires contextual information about its type, the conditions under which it operates, and the collection of measurements. The context information is acquired by filling out a survey, which is further processed to determine the similarity measure. The similarity is calculated concerning other problems analyzed in the past and stored in the database. Each machine analyzed in the past presented on the schematic is represented by a color rectangle. The green color indicates that a given problem from the past has a high similarity value, while red has a low similarity. The several machines analyzed in the past which have achieved the highest similarity value are selected. Each problem contains several solutions proposed during the development of the processing algorithms. These solutions are compared with each other using an efficiency measure. Unique processing algorithms are selected and sorted according to past performance and similarity to the analyzed problem. The several algorithms that achieved the highest score from the comparison are chosen. These algorithms are proposed for the development of a machine condition evaluation algorithm.

The following assumptions are required regarding the set of structures analyzed in the past:

1. The database contains signal processing prescriptions and diagnostic outcomes from many objects with different levels of similarity to the problem at hand. At least some of these similarity levels are very high in meaning, and the very similar problems were already solved.
2. These structures were previously monitored using various signal processing and classification approaches.
3. There exists a description of the structures regarding their design and the conditions under which they operate.

Note that the proposed approach is expected not to provide a completely new set of processing methods but rather to choose a solution that is likely to produce a high outcome in a given scenario. For that reason, the database is required to contain at least some very similar objects. For instance, diagnosing an epicyclic gearbox for a wind turbine would indicate that other epicyclic gearboxes working under similar operational conditions should be available in the reference set.

**Figure 3:**
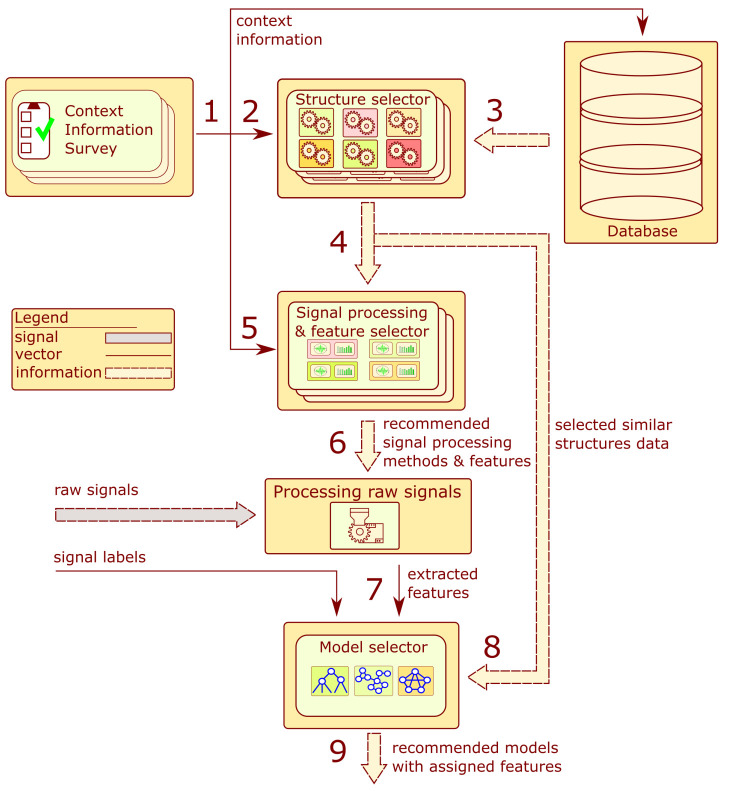
Block diagram of the processing recommendation system.

A more detailed framework of the recommendation system is presented in Figure 3. The system consists of six main components: the context information surveys, database, structure selector blocks, signal processing, feature selection blocks, processing raw signal block, and model selector. The arrows indicate a flow of data within the system. The order of operations performed by the system corresponds to numbers in Figure 3.

In the first step, an adequate survey is completed for context information about the structure. Steps 2 to 6 are performed without human intervention. The context information is employed to calculate a measure of similarity to the structures stored in the database. A list of structures with the highest similarity value is selected. The historical information about signal processing and extracted features from those objects is applied to develop a list of recommended signal processing methods and features.

The signals provided by the user are processed according to the proposed algorithms to extract feature values from signals. The calculated values with the provided labels constitute the training set employed to recommend the model type. The following subsections contain a detailed description of the hinted blocks.

### 3.1. Context Information Survey

The context information is provided utilizing surveys filled out by an operator. The questions in the survey belong to the following thematic categories:

- Variability in operational parameters associated with speed and load variations. This group includes context inputs with the following tags: speed_variability, speed_order, load_variability, load_order.
- The method of conducting the experiment related to the sensor, its location, and the phase marker signal. This group includes context inputs with the following tags: phase_marker, sensors_bandwidth, sensors_location, sensors_direction, sensors_type.
- Type of problem indicating a potentially damaged component and an indicative number of measurement data. This group includes context inputs with the following tags: problem_component, problem_labels, problem_type.

The specific questions depend on the category into which the monitored structure falls. The developed questionnaire can be found in Appendix A.

### 3.2. Structure Selector Module

**Figure 4:**
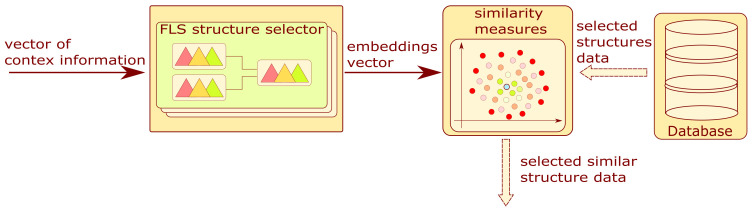
Block diagram of structure selector module.

The block diagram is shown in Figure 4. This module allows for the selection of similar structures. As a result of the completed survey, the context information is quantitatively encoded in a vector.

The mentioned vector is the input to the group of FLSs. Based on context information, one fuzzy system is selected. Each of these systems has four categorized outputs:

- Embedding 1—Variation in operational parameters;
- Embedding 2—Method of experimenting;
- Embedding 3, 4—Type of problem.

The structure data were extracted as a result of the database query. For each selected structure, the similarity value is calculated from the Euclidean measure with the formula presented below:

(1) $$Sim_{i}=\exp\left(-\sqrt{\sum_{n=1}^{4}{\left(em_{n}-em_{nsi}\right)}^{2}}\right)$$

where $Sim_{i}$ is the Euclidean similarity measure, $em_{n},n=1,\ldots ,4$ are the values of the four embeddings calculated using the fuzzy logic system, and $em_{nsi},n=1,\ldots ,4$ are the values of the four embeddings for the *i*-th structure acquired from the database.

The operation allows the system to narrow down the number of structures analyzed in the next step. The final result of this block is a list of structures with similarity measures.

The FLS structure selector is a parallel fuzzy tree where each branch is designated to evaluate the value of an assigned embedding. The architecture of the aforementioned subsystem will be considered in Section 3.2.1.

#### 3.2.1. FLS Structure Selector

**Figure 5:**
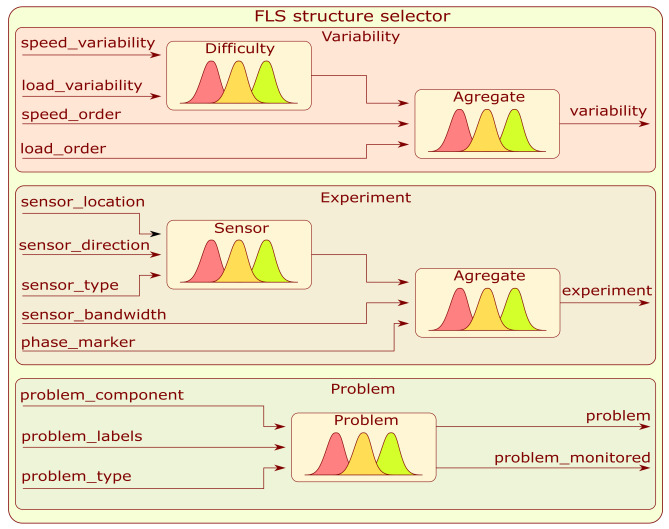
Block diagram of structure selector FLS.

The FLS structure selector has a tree architecture with highlighted branches dedicated to a single embedding value. The first in order is the branch labeled as Variability. This system is a fuzzy tree composed of two fuzzy subsystems in a manner presented in Figure 5. The first Sugeno-type FLS combines information about load with the speed variation to form a hidden variable called difficulty. The second Mamdani FLS combines the calculated hidden variable with load and speed order to determine the embedding value labeled as variability.

The second embedding branch labeled as Experiment is another fuzzy tree with a similar structure to Variability—Figure 5. The first Sugeno subsystem is designed to merge sensor location, direction, and type to form a continuous hidden variable. Based on the extracted hidden variable, sensor bandwidth, and speed information, the second Mamdani subsystem produces an embedding value labeled as experiment.

The last developed subsystem labeled as Problem is dedicated to the type of condition assessment problem and a particular part that is to be monitored. It is composed of a single Mamdani-type FLS with three inputs: problem_component, problem_labels, and problem_type and two outputs: problem and problem_monitored. Information on the number of membership functions and rules depends on the type of survey conducted and the question. Details are provided in Appendix B.

### 3.3. Signal Processing and Feature Selector Module

This module proposes signal processing methods and features. The signal processing methods are represented within the system as signal processing chains which contain algorithms divided into subroutines representing all necessary steps performed on the signal.

For instance, to detect the shaft imbalance, the speed signal needs to be examined for the extraction of the rotating velocity harmonics amplitudes. In the majority of monitoring systems, vibrations are measured as acceleration. To obtain the speed signal, it is necessary to integrate it numerically. This operation results in a trend related to the introduced integration error that can be removed with a high-pass filter or a detrending algorithm. The harmonics analysis is best for representing the signal in the frequency domain by carrying out a digital Fourier transform (DFT). In a derived processing chain, the operation can be expressed as signal detrending–integration–high-pass filtering–DFT.

**Figure 6:**
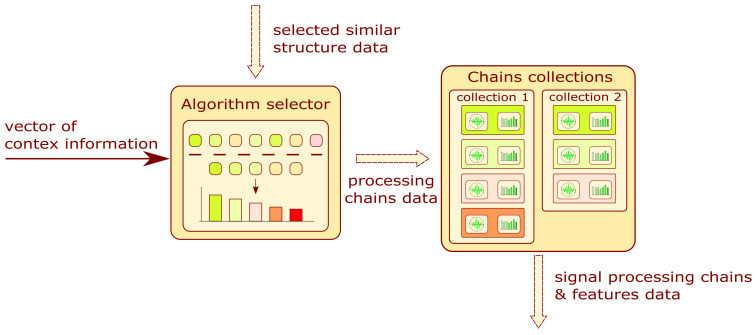
Block diagram of signal processing and feature selector module.

The block diagram of the analyzed module is presented in Figure 6. The input is a vector of context information and selected similar structure data. The algorithm filters the processing chains and features related to a structure by assigning probability values to them. The result of the subsystem is a list of chain collections and features sorted in probability value order. More information on the data filtering algorithm and chain collections can be found in Section 3.4 and Section 3.6.

### 3.4. Algorithm Selector

**Figure 7:**
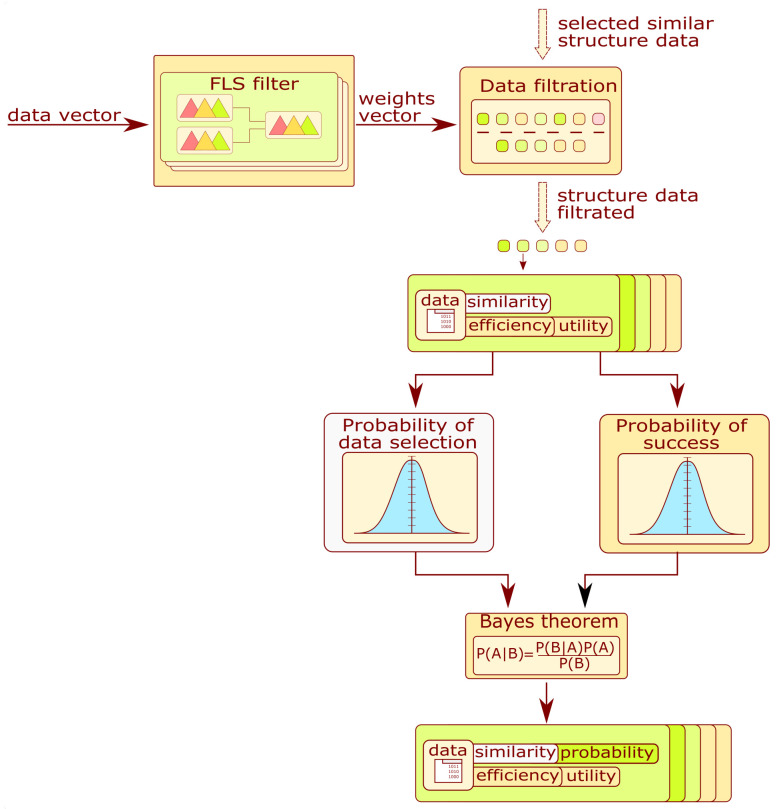
Block diagram of algorithm selector module.

The purpose of this subsystem is the proposition of algorithms applied for signal processing or the assessment of the structure state. The block diagram describing information flow in the module is presented in Figure 7. The fuzzy system is selected and fed with this vector to produce the weight for each signal processing chain or model type. That values are employed to filter structure data obtained by structure selectors (Section 3.2). Each algorithm is assigned the utility value calculated from the weights. The utility value for an algorithm can be expressed by the geometric mean:

(2) $$U_{i}={\left(\prod_{k=1}^{n}w_{k}\right)}^{\frac{1}{n}}$$

where $w_{k}$ is the weight value obtained from the FLS, and *n* is the number of data subentities. For the signal processing and feature selector, *n* is related to the number of methods in the processing chain. For the model selector, the value is set to 1. After these calculations, the system discards the data entities whose utility level is below the threshold of 0.5. As a result of filtration, their number is reduced.

In the next step, the probability of selecting an algorithm given the condition of success is calculated. The algorithm for calculating the coefficient is presented in Figure 7. The similarity value is assigned to the algorithms related to database structures. Based on those values and the number of algorithms, the probability of selecting the algorithm is formulated as

(3) $$P\left(A_{i}\right)=\frac{Sim_{i}}{N_{a}}$$

where $A_{i}$ is the *i*-th algorithm, $Sim_{i}$ is the similarity value for the *i*-th structure for which the algorithm was used, and $N_{a}$ is the number of unique algorithms. Each algorithm contains an attribute called the efficiency coefficient. It describes the result obtained during the structure condition evaluation on the test set. By applying this information and the utility value, the probability of a correct assessment (CA) given the algorithm selection is calculated from the following equation:

(4) $$P\left(CA|A_{i}\right)=\frac{E_{i}}{N_{a}}\cdot U_{i}$$

where $E_{i}$ is the efficiency value of the *i*-th algorithm and $U_{i}$ is the utility value.

The probability of selecting an algorithm given the prior knowledge of a correct assessment can be expressed by introducing the Bayes theorem.

(5) $$P\left(A_{i}|CA\right)=\frac{P\left(A_{i}\right)\cdot P\left(CA|A_{i}\right)}{\sum_{j=1}^{N_{a}}P\left(A_{j}\right)\cdot P\left(CA|A_{j}\right)}$$

As a subsystem result, filtered data entities are sorted in decreasing order of probability. The probability expressed by Equation (5) is expressed for the signal processing and feature selector as $P(Ch_{i})$, $P(CA|Ch_{i})$ and $P(Ch_{i}|CA)$ and for model selectors $P(M_{i})$, $P(CA|M_{i})$, and $P(M_{i}|CA)$.

The fuzzy system configuration for the data filter is a parallel fuzzy tree where a single branch is designated to recommend a single data entity. The architecture of the mentioned tree will be considered in Section 3.4.1 for the signal processing selector and in Section 3.5.2 for the model selector.

#### 3.4.1. Data Filter for Signal Processing Methods

Each signal processing method filter branch is a Mamdani-based fuzzy system dedicated to a single processing method. Each of the mentioned systems contain four inputs. Three of them correspond to three values from the context information described in Section 3.1: phase_marker, sensors_type, and problem_component. The fourth input is the variability value extracted from the model described in Section 3.2.1. The number of Gaussian membership functions is different for each input, but together they cover the whole input range evenly. The output contains five Gaussian membership functions (very_low, low, medium, high, and very_high) that evenly cover the value range from 0 to 1. Information on the construction of fuzzy rules can be found in Appendix C.

### 3.5. Model Selector Module

**Figure 8:**
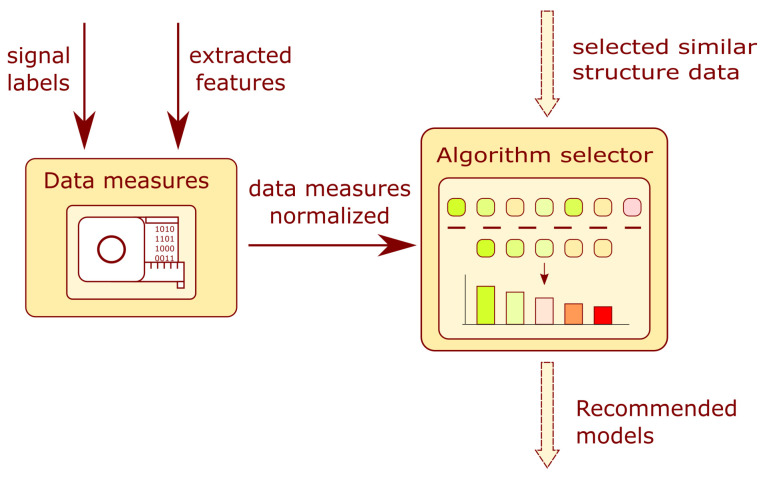
Block diagram of the model selector module.

The module schematic is presented in Figure 8. The module is composed of two main blocks: the data measures and the algorithm selector. The arrows indicate the flow of information in the system. The user provides the system with a prepared training dataset. For the prepared dataset, measures are calculated which describe the data structure. Combining both information forms, the data measures and the similarity of the structures, the data selector determines a list of recommended models applying a data filtering algorithm. More information on the topic can be found in Section 3.4.

#### 3.5.1. Data Measures

This subsystem allows for a quantitative description of the data structure in the training dataset. The input to the block is a vector containing $N_{e}$ training examples of form $\left\{\left(x_{1},l_{1}\right),\ldots ,\left(x_{N},l_{N}\right)\right\}$ such that $x_{i}$ is the feature vector and $l_{i}$ is the target label of the *i*-th sample. The output of the subsystem is a vector of the following measures:

1. The number of training examples—$N_{e}$;
2. The number of input features—$N_{f}$;
3. The number of output target labels—$N_{t}$;
4. The measure of a non-uniform spread of data among clusters—$S_{t}$;
5. The clusters-to-targets ratio—$R_{ctr}$;
6. The clusters’ mean diversity—$D_{cmd}$.

All extracted measures from the training set are subject to normalization.

The measure of a non-uniform spread of data among the clusters coefficient is calculated as a standard deviation of a class presence probability. The feature allows for measuring whether the training samples are uniformly distributed among classes or whether some classes are under-represented. It is calculated using the following formula:

1. The probability of a selection sample for given target classes from the training set is calculated.
2. From the derived set of probabilities, the standard deviation is extracted with the following standard formula:
  (6) $$S_{t}=\sqrt{\frac{1}{N_{t}-1}\sum_{i=1}^{N_{t}}{\left(p_{Ct_{i}}-\bar{p_{Ct}}\right)}^{2}}$$
  where $N_{t}$ is the number of target classes, $p_{Ct_{i}}$ is the probability of sample selection for a given target class $Ct_{i}$ from a training set, and $\bar{p_{Ct}}$ is the mean probability.

The clusters-to-targets ratio is defined by following equation:

(7) $$R_{ctr}=\frac{N_{cl}}{N_{t}}$$

where $N_{cl}$ is the number of the selected clusters by algorithm and $N_{t}$ is the number of target classes. The $N_{cl}$ value is obtained by performing the following algorithm:

1. Estimate four optimal numbers of clusters by using four different cluster evaluation criteria:
  - The Calinski–Harabasz criterion;
  - The Davies–Bouldin criterion;
  - The Silhouette value criterion;
  - The Gap value criterion.
  For each criterion, the optimal cluster value is stored in the memory. A more detailed description of the listed criteria can be found in the literature [33].
2. From the calculated collection of optimal cluster amounts, the most frequent value is selected and assigned as the cluster number $N_{cl}$.

Clusters’ mean diversity measures target variability within clusters. The algorithm for the described feature contains the following steps:

1. The input feature space is divided into an optimal number of clusters ($N_{cl}$).
2. For each cluster, the probability of selecting a sample belonging to a given class ($p_{Ct_{i}}$) is calculated.
3. The dominant class with the highest probability calculated in the previous step is selected.
4. The probability of selecting a sample from the other classes is calculated according the following relation:
  (8) $$P\left(∼D|Cl_{i}\right)=1-P\left(D|Cl_{i}\right)$$
  where $P(D|Cl_{i})$ is the probability of selecting a sample from the dominant class *D* given the *i*-th cluster $Cl_{i}$.
5. Steps 3 and 4 are repeated until the probability $P(∼D|Cl_{i})$ is calculated for all defined clusters.
6. The cluster mean diversity is obtained from the following equation:
  (9) $$D_{cmd}=\sum_{i=1}^{N_{cl}}w_{i}P\left(∼D|Cl_{i}\right)$$
  where $N_{cl}$ is the optimal number of clusters, and $w_{i}$ is the weight factor for the *i*-th cluster, defined by:
  (10) $$w_{i}=\frac{N_{eCli}}{N_{e}}$$
  where $N_{eCli}$ is the number of examples belonging to the *i*-th cluster and $N_{e}$ is the number of training examples.

#### 3.5.2. Data Filter for Model Type

**Figure 9:**
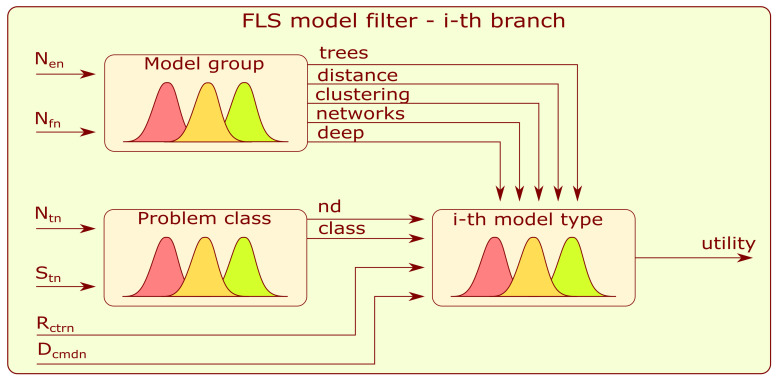
Block diagram of the branch of the model filter.

Figure 9 presents a detailed block diagram of the selected *i*-th branch of model filter FLS. Each branch is composed of three subsystems: model group, problem class, and ith type of model.

Each FLS mentioned in this subsection is of the Mamdani type. The first to be described is the model-group FLS. Its five outputs are related to the following groups of algorithms: trees, distance-based, clustering-based, neural networks, and deep learning. The inputs to the subsystem are a normalized number of training examples and a number ($N_{en}$) of input features ($N_{fn}$). Each input and output contains five Gaussian membership functions (very_low, low, medium, high, and very_high) that evenly cover the value range from 0 to 1.

The FLS problem class has a similar structure to the first-mentioned model. The novelty detection (nd) and classification (class) outputs refer to types of machine learning. The inputs to the subsystem are normalized values of the output target’s labels number ($N_{tn}$) and the standard deviation of target classes ($S_{tn}$). Five Gaussian-type membership functions are assigned for input fuzzification.

The *i*-th type of model combines information from the above-mentioned submodels and the additional normalized inputs: the clusters to targets ratio ($R_{ctrn}$) and clusters mean diversity ($D_{cmdn}$)—Figure 9. The fuzzification and defuzzification procedures are similar to those for the two models described above, and five Gaussian-type membership functions are applied. The rules developed for the model filter can be found in Appendix D.

### 3.6. Chain Collections

The recommended processing chains and features with assigned similarity, efficiency, and probability values are grouped into chain collections. The collections are prepared based on information about features used for training the recommended models. After preparing the collection, the algorithm discriminates in terms of its utility. The equation for this measure, for the n-th collection, is the following:

(11) $$U\left(Col_{n}\right)=\max_{1\leq i\leq N_{ch}}\left\{E_{i}\right\}\max_{1\leq i\leq N_{ch}}\left\{Sim_{i}\right\}\sum_{i=1}^{N_{ch}}P\left(Ch_{i}|CA\right)$$

where $\max_{1\leq i\leq N_{ch}}\left\{E_{i}\right\}$ is the maximum value of efficiency obtained for the collection, $\max_{1\leq i\leq N_{ch}}\left\{Sim_{i}\right\}$ is the maximum similarity value obtained for the collection, and $\sum_{i=1}^{N_{ch}}P\left(Ch_{i}|CA\right)$ is the sum of probabilities obtained for chains included in the collection. From the utility value $U(Col_{n})$, a probability value of the collection recommendation is calculated, according to the following formula:

(12) $$P\left(Col_{n}\right)=\frac{U(Col_{n})}{\sum_{n=1}^{N_{col\_ch}}U\left(Col_{n}\right)}$$

where $N_{col\_ch}$ is the number of the collections.

## 4. Implementation of the Recommendation System

**Figure 10:**
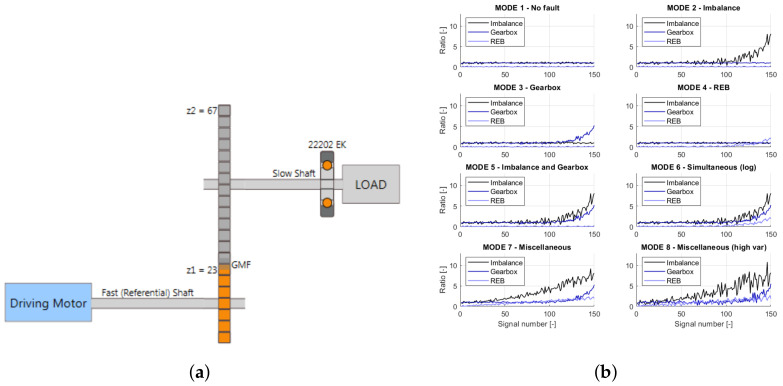
(**a**) Simulated object kinetostatic scheme [34]; (**b**) failure development functions for a nominal velocity of 3000 rpm [34].

The framework was used to develop the system for parallel gearboxes. This involved developing a database of historically monitored gears. As a monitored structure, the object representing a simulated drivetrain was utilized. It included a driving shaft associated with referential speed, a one-stage gearbox, a slow shaft, and a rolling element bearing (REB) 22202EK. The gearbox was used for speed reduction, with 23 teeth on the driving shaft gear and 67 teeth on the slow shaft gear. The total transmission ratio was 23/67 = 0.34328. The kinetostatic diagram of the simulated object is presented in Figure 10a.

The simulated object operates at several nominal speeds from 2400 to 6000 rpm, with a different speed fluctuation from around 3 to 3000 rpm. The simulated data represent eight modes of object structural failures. The list of existing failure modes is presented in Table 1. Each failure mode is represented by the failure development function (FDF) that determines the particular fault evolution. For vibrational signal generation in the failure mode, the simulated object requires three FDFs, which indicate the shaft, gearbox, or bearing faults. The sample FDFs for a nominal velocity of 3000 rpm are presented in Figure 10b. Each mode contains 150 independent vibrational signals, a 10 s time window, and a sampling frequency of 25 kHz. In addition to the vibration signals, a phase marker signal was generated for half of the objects. More information about the simulated object itself and the algorithms behind signal generation can be found in [34].

### 4.1. Signal Processing and Feature Extraction

The prepared dataset was processed to extract indicators sensitive to the presence of damage. During years of research related to vibrodiagnostics, many signal processing and feature extraction algorithms have been proposed, from the fundamental calculation of the raw-signal root mean square (RMS) to more sophisticated spectral kurtosis. A detailed description of various processing algorithms dedicated to CM can be found in [34,35]. Based on the information in the literature and the visual inspection of the indicators, the signals were processed. A detailed list of the signal processing algorithms and the extracted features is collected in Table 2.

### 4.2. Trained Models

For the calculated features and labels stored in the database, a training set was prepared. The final goal of this operation was to obtain a quantitative assessment of the extracted features by training multiple models. These algorithms were diversified in terms of type and operations related to the clusters division of the training set, distances, or trained weights. The list of model types and their parameters are presented in Table 3.

### 4.3. Test Structures

To evaluate the developed system, two datasets for parallel gearboxes were prepared. The first dataset was obtained for the simulated object of a one-stage gearbox (Gearbox 1), the schematic for which is presented in Figure 10a. The vibration signals were acquired for eight fault modes presented in Figure 10b, where the object worked at a nominal rotational velocity of 4200 rpm with 1020 rpm fluctuations, not present in the database. The 400 signals with phase markers were generated to construct the test set. For this structure, labels were designated based on the failure development function values presented in Figure 10b. If the function values exceeded those for mode 1 (Table 1), a label was assigned to the signal. In total, a set of six different labels describing the following states of the structure was prepared: G1C1, undamaged; G1C2, imbalance; G1C3, gear meshing fault; G1C4, bearing fault; G1C5, imbalance and gear meshing fault; and G1C6, imbalance, gear meshing fault, and bearing fault.

**Figure 11:**
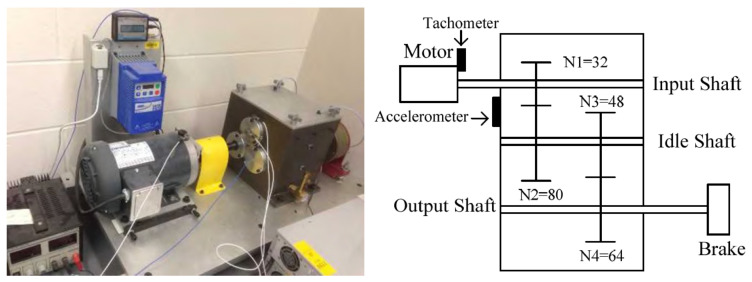
Two-stage gearbox picture (**left**) and kinematic diagram (**right**) [36].

The second dataset was obtained for a two-stage gearbox (Gearbox 2), the schematics for which are presented in Figure 11. The signals were acquired for multiple faults, such as missing tooth, root crack, spalling, and chipping tip with five different levels of severity. The data were recorded in the angle domain with 900 samples per revolution. The dataset was first presented in [36] and is available for free. The inputs vector for the analyzed gearboxes and vectors for similar structures are stored in Table 4. For Gearbox 2, the labels were assigned directly from the measurement series names. In total, the set of five different labels describe the following states of the structure: G2C1, undamaged; G2C2, missing tooth; G2C3, cracked tooth root; G2C4, spalled tooth surface; and G2C5, chipping tooth tip.

### 4.4. Recommendation Algorithms Accuracy

To evaluate recommendation algorithms, two metrics were proposed: class accuracy and total accuracy. The class accuracy is defined as:

(13) $$GiCj=\frac{N_{cor-class}}{N_{samp-class}}$$

where $N_{cor-class}$ is the number of correct model predictions within the class in the test set, $N_{samp-class}$ is the total number of samples belonging to the mentioned class in the test set, and GiCj is the class accuracy. The formula for the second measure is presented by the following equation:

(14) $$GiA=\frac{N_{cor}}{N_{samp}}$$

where $N_{cor}$ is the number of correct model predictions in the test set, $N_{samp}$ is the total number of samples in the test set, and GiA is the accuracy value. For an unbiased selection of the test set, cross-validation was performed. The procedure contained 30 iterations, while each model was trained 10 times by randomly selecting 10% of the samples for the test set.

## 5. Results and Discussion

### 5.1. Recommended by System Signal Processing Methods and Models

The first recommendation result is a list of signal processing chains with features. For an object named Gearbox 1, the system recommended six different processing chains. Gearbox 2 was proposed with four processing recommendations. A detailed list of all processing chains and features is presented in Table 5. The processing chains are sorted by decreasing probability according to Equation (5).

All of the proposed processing chains include the spectrum calculation. The possible gearbox faults related to gears or bearings reveal its presence as the harmonics of driving speed. Their amplitude makes a minor contribution to the total response signal. The spectrum analysis increases the probability of detecting damage at an early stage of its development. Thus, the first recommended processing chain is a spectrum analysis in the angle domain. Chain B is extracted from time signal phase-locked components. This operation is convenient for the detection of frequency components linked with gear meshing and rolling bearing failures [34]. The third-order proposed chain obtains the velocity from the acceleration signal. Such an analysis allows for the detection of shaft damage, manifested in low frequencies [35]. Chain D is dedicated to the calculation of the two features from the envelope spectrum from the resampled signal. In the literature [34], envelope analysis was found to be convenient for detecting cyclostationary components generated by faulty rolling-element bearings and gear meshing. The last two presented processing chains (E and F) do not have a firm background in the CM literature. The first is focused on detecting gear-fault-related components. The second is related to the extraction of instantaneous velocity.

Upon analyzing the second part of Table 5 with the recommended chains for Gearbox 2, it is evident that the C chain selection was repeated. This time it is recommended as the last one due to its being less effective in detecting the meshing damage that analysis in Gearbox 2 is dedicated to. The first in order chain for Gearbox 2 is similar and represents the frequency spectrum analysis, elementary for time domain signals. The following chain is envelope extraction, the function of which is described above in this subsection. The last described in this paragraph is the I chain related to the straightforward extraction of statistical features from the time domain signal.

The collected signals for both structures were processed using the processing chains proposed by the system presented in Table 5. A set of labels described in Section 4.3 was assigned to the designated collection of features to form training sets. For model recommendation, the prepared sets were used to calculate the features described in Section 3.5.1. Based on the values and data determined for similar structures, the system proposed a list of models with a proposed set of features included in Table 6 for each analyzed gearbox. The algorithm for calculating the probability is described in Section 3.4. According to the produced recommendations, all models obtained the best results for the data classification with a low number of dimensions. These models had hyper-parameter values based on information stored in a database and extracted for similar structures. Referring to the table, these are:

- Random forest: $N_{trees}=20$, $N_{minto}=1$;
- Decision tree: $N_{maxds}=20$, $N_{minlo}=1$;
- kNNC: k=5, s=false.

**Table 6:** Models recommended for Gearbox 1 and Gearbox 2.

Model Type	Features	Success Rate	Probability
Recommended for Gearbox 1			
MG11: random forest	A, B, C, D, E, F	0.96	0.296
MG12: decision tree	A, B, C, D	0.97	0.259
MG13: random forest	A, B, C, E, F	0.97	0.246
MG14: random forest	A, B, C	0.98	0.199
Recommended for Gearbox 2			
MG21: random forest	G, H, I, C	0.96	0.31
MG22: kNNC	G, H, I	0.91	0.27
MG23: random forest	G, H, C	0.97	0.23
MG24: kNNC	G, H	0.91	0.19

### 5.2. Non-Recommended Signal Processing Methods and Models

To validate the recommendations produced by the system, an additional list of methods and models was prepared. The proposed algorithms were selected from the list contained in Table 2 and Table 3 by considering only the non-recommended ones. Thus, for Gearbox 1, processing methods were chosen. They are related in not containing a signal resampling procedure but still including a spectrum analysis. The processing methods selected for Gearbox 2 assume signal processing in the time domain, utilizing envelope extraction. The list of processing methods is stored in Table 5. Additionally, the models were specified in a manner that was not proposed by the system. Two types were selected: MLP and kMC. The hyper-parameter values for those models are the following:

- MLP: L=5,
- kMC: k=20.

### 5.3. Signal Processing Methods and Models Evaluation

**Figure 12:**
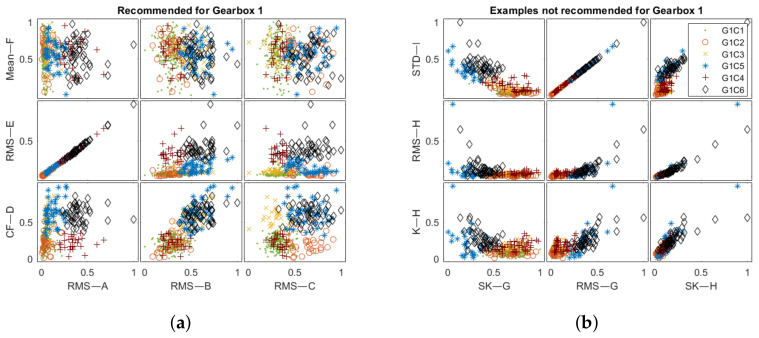
The feature space obtained for the Gearbox 1 test set for: (**a**) recommended and (**b**) not recommended processing chains.

**Figure 13:**
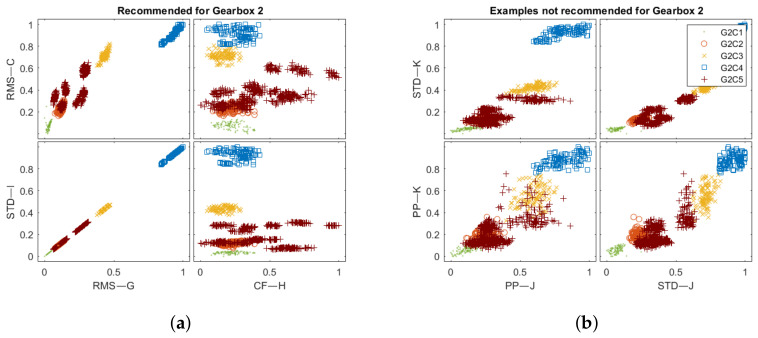
The feature space obtained for Gearbox 2 test set for: (**a**) recommended and (**b**) not recommended processing chains.

Figure 12 and Figure 13 reveal the training set in feature space obtained from the chains in Table 5. The number of indicators was reduced to allow for visual result comparison. The extracted features for the recommended processing chains allow the presented clusters to be more separable. For the Gearbox 1 recommended features (Figure 12a), the points cover the available normalized space more evenly. On the other hand, not recommended feature values are tight to compose more mixed clusters (Figure 12b). For the Gearbox 2 dataset (Figure 13), the difference between recommended and not recommended is not evident at a glance. Both processing chain collections produce feature space with good cluster separation.

**Figure 14:**
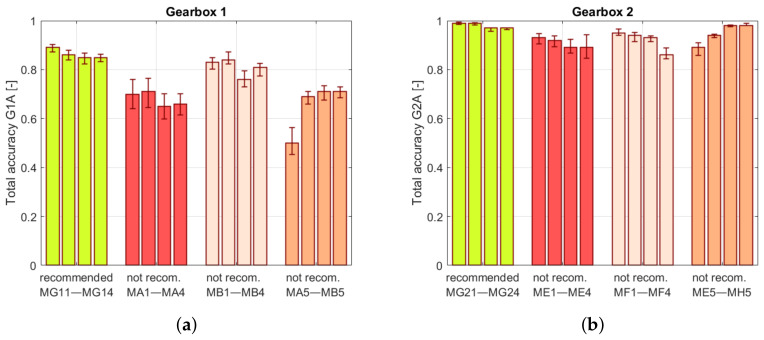
Total accuracies obtained for models trained on the dataset obtained for (**a**) Gearbox 1 and (**b**) Gearbox 2. The whiskers indicate minimal and maximum accuracy values.

The results obtained for trained models in Table 7 and Figure 14a reveal that the algorithms proposed by the system have a higher accuracy in comparison to the non-recommended ones. This includes both overall and class accuracy. This difference is most noticeable in the classes for which non-recommended models have the lowest accuracy value. The lowest accuracy values were obtained for the G2C2 class. The best-recommended model scored 0.56, while others for the same set of features were 0.48 (KMC) and 0.43 (MLP). The percentage difference was approximately equal to 14% and 23%. The highest accuracy was achieved by the trained models for the G1C1 class labeled as the healthy state. The high rate of correct labels in this class indicates a potentially lower number of false alarms. In this category, the difference between the recommended models is negligible and, in the worst case, equal to 0%. The model accuracy obtained for non-recommended features reveals a significant difference. For the G1C2 class, the models had a low accuracy equal to 0 for KMC. Only for the G1C1 class did this value reach a reasonable level, although it was still lower than that for all models and features proposed by the system.

The results of the recommendation system tests for Gearbox 2 are stored in Table 8 and Figure 14b. The accuracies obtained for the recommended types of models reveal a higher level of effectiveness. This applies to accuracy in individual classes as well as in total. Comparing the results for the G2C2 class, the greater accuracy of the recommended models is visible. For the set of MG21 features, the recommended model achieved an accuracy of 0.93, and the non-recommended ones were KMC 0.78 and MLP 0.7. The percentage differences in accuracy were 16% and 25%. For the other classes, the accuracy was higher, and the relative difference did not exceed 10%. For the non-recommended processing chain results, there was no significant difference between the accuracies. The accuracy for MLP and KMC models was significantly lower. However, the models proposed by the system achieved results on a similar level as those obtained for the recommended features.

## 6. Summary and Conclusions

This article presents a processing recommendation system for condition monitoring based on fuzzy logic. The system structure is knowledge-based, and the recommendations are proposed based on contextual information. The main part of the proposed system is a database in which historical data from previously analyzed structures are stored. Based on calculated embedding values, similar objects are selected. The obtained data are further filtered by subsequent subsystems for better adjustment to the analyzed problem.

The article contains an implementation of the framework and a test of the presented system by processing data for two structures. The first is virtual objects that simulate signals collected from a single-stage parallel gearbox. The second is a two-stage gearbox. The system recommended processing algorithms, which were then compared with others that were not recommended, selected arbitrarily. All algorithms were applied for processing data from test objects. The obtained results reveal the feature space and classifier accuracies. The results presented prove that the processing algorithms proposed by the system showed, on average, a from 5 to 14.5% higher accuracy according to all proposed metrics. It indicates that the recommender system structure can produce valuable processing recommendations which facilitate the condition evaluation process.

The proposed recommendation system also contains limitations. The processing algorithms were selected from those available in the database. Thus, the problem solution is not necessarily optimal in global terms. However, by increasing the amount of historical data from structures operating under various conditions, the recommended result will be closer and closer to the optimal solution.

## Figures and Tables

**Table 1:** Failures modes of generated data.

Mode No.	Mode Name	Mode Description
1	No fault	No fault in the entire drivetrain
2	Imbalance	Logarithmic development of the drive shaft imbalance
3	Gearbox	Logarithmic development of the general transmission fault
4	REB	Logarithmic development of the REB inner race fault
5	Imbalance and Gearbox	Simultaneous development of the drive shaft imbalance and the transmission fault
6	Simultaneous	Simultaneous development of all the considered faults
7	Miscellaneous	Simultaneous development of all the faults with different functions
8	Miscellaneous (high var.)	Simultaneous development of all the faults with different functions and high variance

**Table 2:** Signal processing methods and extracted features.

No.	Signal Processing Methods Chain	Extracted Features
1	Linear detrending–signal resampling–spectrum	RMS, skewness (SK)
2	Linear detrending–signal resampling–bandpass filtration (35, 55)–spectrum	Crest factor (CF), kurtosis (K)
3	Linear detrending–signal resampling–bandpass filtration (35, 55)–signal demodulation– spectrum	CF, K, SK
4	Linear detrending–signal resampling–signal envelope–linear detrending–spectrum	RMS, SK
5	Linear detrending–integration–highpass filtration (10 Hz cutoff)–spectrum	RMS
6	Linear detrending–time synchronous analysis–spectrum	Peak to peak (PP), RMS
7	Linear detrending–instantaneous frequency from phase marker signal	Mean
8	Linear detrending–spectrum	RMS, SK
9	Linear detrending–signal envelope–linear detrending–spectrum	CF, SK, K
10	Linear detrending	PP, standard deviation (STD)
11	Linear detrending–bandpass filtration (1000, 10,000 Hz cutoff)–signal envelope–highpass filtration (10 Hz cutoff)–spectrum–linear detrending	CF, K
12	Linear detrending–highpass filtration (10 Hz cutoff)–integration	PP, STD
13	Linear detrending–signal envelope–linear detrending	PP, STD
14	Linear detrending–demodulation–spectrum	CF, K, Shannon entropy (SE)

**Table 3:** Model types and parameters.

Model Type	Parameters	Symbol
Decision tree	Minimum number of leaf node observations	$N_{minlo}$
	Maximal number of decision splits	$N_{maxds}$
Gaussian mixture model classifier (GMMC)	Number of components	*k*
K-means classifier (KMC)	Number of components	*k*
K-nearest neighbor classifier (kNNC)	Number of nearest neighbors	*k*
	Standardize	*s*
Multilayered perceptron (MLP)	Number of neurons in hidden layers	*L*
Random forest	Number of trees	$N_{trees}$
	Minimum number of observations per tree leaf	$N_{minto}$
Radial-based function neural network (RBFNN)	Mean-squared error goal	MSE
	Spread of radial basis functions	Sp
	Maximum number of neurons	$N_{n}$
	Number of neurons to add between iterations	$N_{it}$
Self-organizing map (SOM)	Output layer dimensions	*L*
	Number of training steps for initial covering of the input space	$N_{intcov}$
	Initial neighborhood size	$k_{init}$
	Layer topology function	F(L)
	Neuron distance function	F(D)

**Table 4:** Context information for the acquired data.

Question Tag	Gearbox 1	Similar Database Objects		Gearbox 2	Similar Database Objects		Ranges
speed_variability	3	3	2	1	1	0	[0, 4]
load_variability	1	0	0	1	0	0	[0, 4]
speed_order	0.23	0.3	1	0	0	0	[0, 3]
load_order	0	0	0	0	0	0	[0, 3]
phase_marker	1	1	1	0	0	0	[0, 1]
sensors_bandwidth	25	25	25	0.9	25	25	[1, 100]
sensors_location	1	1	1	0	1	1	[0, 2]
sensors_direction	1	1	1	0	1	1	[0, 2]
sensors_type	0	0	0	0	0	0	[0, 2]
problem_component	4	4	4	1	1	4	[0, 4]
problem_labels	2	2	2	2	2	2	[0, 2]
problem_type	1	1	1	1	1	1	[0, 3]
similarity value	-	0.93	0.79	-	0.69	0.59	[0, 1]

**Table 5:** Signal processing methods and features recommended for Gearbox 1 and Gearbox 2.

Signal Processing Methods Chain	Features	Probability
Recommended for Gearbox 1		
A: linear detrending–signal resampling–spectrum	RMS-A, SK-A	0.19
B: linear detrending–time synchronous averaging–spectrum	PP-B, RMS-B	0.19
C: linear detrending–integration–highpass filtration (10 Hz cutoff)–spectrum	RMS-C	0.18
D: linear detrending–signal resampling–bandpass filtration (35 to 55 1/revolution)–signal demodulation–spectrum	CF-D, K-D, SK-D	0.175
E: linear detrending–signal resampling–signal envelope–linear detrending–spectrum	RMS-E, SK-E	0.155
F: linear detrending–instantaneous frequency from phase marker signal	Mean-F	0.11
Examples not recommended for Gearbox 1		
G: linear detrending–spectrum	RMS-G, SK-G	-
H: linear detrending–signal envelope–linear detrending–spectrum	SK-H, K-H, RMS-H	-
I: linear detrending	PP-I, STD-I	-
Recommended for Gearbox 2		
G: linear detrending–spectrum	RMS-G, SK-G	0.28
H: linear detrending–signal envelope–linear detrending–spectrum	SK-H, K-H, CF-H	0.28
I: linear detrending	PP-I, STD-I	0.25
C: linear detrending–highpass filtration (10 Hz cutoff)–integration–spectrum	RMS-C	0.19
Examples not recommended for Gearbox 2		
J: linear detrending–highpass filtration (10 Hz cutoff)–integration	PP-J, STD-J	-
K: linear detrending–signal envelope–linear detrending	PP-K, STD-K	-

**Table 7:** Model accuracy for Gearbox 1.

Model Type	Features	Accuracy for Classes (G2C1–G2C6) and for All (G1A)						
		G1C1	G1C2	G1C3	G1C4	G1C5	G1C6	G1A
Recommended for Gearbox 1								
MG11: random forest	A, B, C, D, E, F	0.97	0.56	0.74	0.85	0.93	0.94	0.89
MG12: decision tree	A, B, C, D	0.97	0.55	0.74	0.83	0.92	0.94	0.86
MG13: random forest	A, B, C, E, F	0.95	0.54	0.53	0.83	0.88	0.93	0.85
MG14: random forest	A, B, C	0.94	0.53	0.53	0.82	0.89	0.92	0.85
**Not recommended model types**								
MA1: MLP	A, B, C, D, E, F	0.87	0.43	0.46	0.54	0.51	0.59	0.7
MA2: MLP	A, B, C, D	0.88	0.42	0.54	0.54	0.53	0.61	0.71
MA3: MLP	A, B, C, E, F	0.82	0.47	0.33	0.48	0.43	0.54	0.65
MA4: MLP	A, B, C	0.82	0.5	0.37	0.46	0.43	0.57	0.66
MB1: KMC	A, B, C, D, E, F	0.96	0.48	0.65	0.58	0.86	0.87	0.83
MB2: KMC	A, B, C, D	0.97	0.52	0.67	0.61	0.89	0.86	0.84
MB3: KMC	A, B, C, E, F	0.94	0.3	0.26	0.57	0.81	0.85	0.76
MB4: KMC	A, B, C	0.96	0.4	0.41	0.68	0.85	0.86	0.81
Not recommended processing chains								
MA5: MLP	G, H, I	0.68	0.31	0.37	0.22	0.19	0.38	0.5
MB5: KMC	G, H, I	0.94	0	0.12	0.44	0.6	0.8	0.69
MC5: random forest	G, H, I	0.91	0.07	0.41	0.5	0.61	0.81	0.71
MD5: decision tree	G, H, I	0.88	0.14	0.48	0.51	0.61	0.81	0.71
Number of samples in each class		212	34	32	46	27	49	400

**Table 8:** Model accuracy for Gearbox 2.

Model Type	Features	Accuracy for Classes (G2C1–G2C5) and for All (G2A)					
		G2C1	G2C2	G2C3	G2C4	G2C5	G2A
Recommended for Gearbox 1							
MG21: random forest	G, H, I, C	0.99	0.93	0.99	1	0.99	0.99
MG22: kNNC	G, H, I	0.99	0.9	0.99	1	0.99	0.99
MG23: random forest	G, H, C	0.99	0.86	0.99	0.99	0.98	0.97
MG24: kNNC	G, H	0.99	0.85	0.98	0.99	0.97	0.97
Not recommended models types							
ME1: MLP	G, H, I, C	0.92	0.72	0.91	0.96	0.94	0.93
ME2: MLP	G, H, I	0.92	0.77	0.89	0.98	0.93	0.92
ME3: MLP	G, H, C	0.91	0.65	0.86	0.95	0.92	0.89
ME4: MLP	G, H	0.93	0.6	0.86	0.96	0.92	0.89
MF1: KMC	G, H, I, C	0.99	0.78	0.99	0.99	0.96	0.95
MF2: KMC	G, H, I	0.96	0.71	0.98	0.99	0.96	0.94
MF3: KMC	G, H, C	0.98	0.72	0.99	0.99	0.94	0.93
MF4: KMC	G, H	0.6	0.4	0.98	1	0.93	0.86
Not recommended processing chains							
ME5: MLP	J, K	0.96	0.54	0.94	0.99	0.90	0.89
MF5: KMC	J, K	0.98	0.78	0.93	0.99	0.95	0.94
MG5: kNNC	J, K	0.98	0.93	0.97	0.99	0.97	0.98
MH5: random forest	J, K	0.99	0.92	0.99	0.99	0.98	0.98
Number of samples in each class		104	104	104	104	520	936

## Data Availability

Data used in this study are available upon request from the corresponding author.

## Abbreviations

The following abbreviations are used in this manuscript:

CM	Condition monitoring
BT-SVM	Binary tree support vector machine
FLS	Fuzzy logic system
PBSHM	Population-based structural health monitoring
DFT	Discrete Fourier transform
FDF	Failure development function
REB	Rolling element bearing
RMS	Root mean square
SK	Skewness
CF	Crest factor
K	Kurtosis
PP	Peak to peak
STD	Standard deviation
SE	Shannon entropy
GMMC	Gaussian mixture model classifier
KMC	K-means classifier
KNNC	K-nearest neighbor classifier
MLP	Multilayer perceptron
RBFNN	Radial-based function neural network
SOM	Self-organizing map

## Appendix A. Questions Developed for the Survey

The questions developed to obtain contextual information about the structure are presented below.

1. Variability of operational parameters
  - speed_variability: What is the speed variation?
    - 0—Constant, same as in the new device.
    - 1—Constant, other than in the new device.
    - 2—Variable but constant during measurements.
    - 3—Variable, small fluctuations during measurements.
    - 4—Variable, changes during signal acquisition in the whole range.
  - speed_order: By what order of magnitude does the speed change?
    - Real number from 0 to 3.
  - load_variability: What is the load variation?
    - 0—Constant, same as in the new device.
    - 1—Constant, other than in the new device.
    - 2—Variable but constant during measurements.
    - 3—Variable, small fluctuations during measurements.
    - 4—Variable, changes during signal acquisition in the whole range.
  - load_order: By what order of magnitude does the load change? Answer:
    - Real number from 0 to 3.
2. The method for conducting the experiment
  - phase_marker: Is there a phase marker signal available?
    - 0—No.
    - 1—Yes.
  - sensors_bandwidth: What is the sensor bandwidth (kHz)?
    - Real number from 0.5 to 1000.
  - sensors_location: What is the location of the sensors?
    - 0—On the housings.
    - 1—On the bearings.
    - 2—Both locations.
  - sensors_direction: What is the direction of sensor measurements?
    - 0—Parallel to rotational axis.
    - 1—Perpendicular to rotational axis.
    - 2—Both directions.
  - sensors_type: What is the type of sensors?
    - 0—Accelerometer.
    - 1—Vibrometer.
    - 2—Both types.
3. Type of problem
  - problem_component: Which component will be monitored?
    - 0—Bearing.
    - 1—Gear.
    - 2—Shaft.
    - 3—Other.
    - 4—Multiple components.
  - problem_labels: How many examples of fault states are there for the monitored component?
    - 0—None.
    - 1—Few examples.
    - 2—Multiple examples.
  - problem_type: What is the type of the problem?
    - 0—Fault detection.
    - 1—Assessment of the condition.
    - 2—Assessment of the temporal condition.
    - 3—Multiple problems.

## Appendix B. Developed Rules for the Structure Selector

The difficulty system is a Sugeno-type system. Each of its inputs contains five Gaussian membership functions that correspond to the speed_variability and load_variability answers presented in Appendix A. The output also contains the same number of membership functions in a range from 0 to 1 with the following names: very_low, low, medium, high, and very_high. Based on membership function sets, the five rules were extracted. The membership function parameters were tuned to fit the values presented in Table A1. The values in the table were filled in based on a survey performed with industrial practitioners. The algorithm used was gradient descent with gradient calculated using the backpropagation algorithm widely employed in neural networks training.

**Table A1:** Look-up table for Difficulty system development.

		Load_Variability				
		0	1	2	3	4
speed_variability	0	0	0.05	0.2	0.4	0.7
	1	0.05	0.08	0.2	0.5	0.9
	2	0.2	0.22	0.3	0.6	1
	3	0.4	0.45	0.6	0.7	1
	4	0.7	0.8	0.85	0.9	1

The Aggregate subsystem of the Variability branch contains the Mamdani structure. The speed_order and load_order inputs contain three Gaussian-type membership functions (low, medium, and high) which overlap the input values evenly. The output from the Difficulty subsystem and the variability output contains five membership functions (very_low, low, medium, high, and very_high). The developed rules for the system are presented below in the form of a list:

- IF Difficulty IS very_low THEN variability IS very_low;
- IF Difficulty IS low THEN variability IS low;
- IF Difficulty IS medium THEN variability IS medium;
- IF Difficulty IS high THEN variability IS high;
- IF Difficulty IS very_high THEN variability IS very_high;
- IF speed_order IS low THEN variability IS very_low;
- IF speed_order IS medium THEN variability IS medium;
- IF speed_order IS high THEN variability IS very_high;
- IF load_order IS low THEN variability IS very_low;
- IF load_order IS medium THEN variability IS medium;
- IF load_order IS high THEN variability IS very_high.

The Sensor fuzzy subsystem is a Sugeno-type subsystem. Each of its inputs contains three Gaussian membership functions that correspond to the sensors_location, sensors_direction and sensors_type answers presented in Appendix A. The output also contains the same number of membership functions (very_difficult, and very_convenient) in a range from 0 to 1. The two rules were obtained by tuning the system to values from lookup Table A2 using the same algorithm as was used for the Difficulty subsystem. The values in the table were also filled in based on the survey performed with industrial practitioners.

**Table A2:** Look-up table for Sensor system development.

		Sensor_Direction|Sensor_Type								
		0|0	0|1	0|2	1|0	1|1	1|2	2|0	2|1	2|2
sensor_location	0	0.93	0.93	0.93	0.8	0.8	0.8	0.5	0.5	0.5
	1	0.78	0.78	0.78	0.67	0.64	0.64	0.36	0.35	0.36
	2	0.47	0.47	0.47	0.33	0.33	0.33	0.1	0.1	0.1

The Aggregate subsystem of Experiment branch architecture is the Mamdani. The sensor_bandwidth input contains five Gaussian-type membership functions which overlap input values evenly. The speed_information contains two Gaussian membership functions corresponding to answers from Appendix A. The Sensor input and Experiment output contain five membership functions. The system rules are stored in the following list:

- IF Sensor IS very_low THEN Experiment IS very_convenient;
- IF Sensor IS low THEN Experiment IS convenient;
- IF Sensor IS medium THEN Experiment IS medium;
- IF Sensor IS high THEN Experiment IS difficult;
- IF Sensor IS very_high THEN Experiment IS very_difficult;
- IF speed_information IS Yes THEN Experiment IS very_convenient;
- IF speed_information IS No THEN Experiment IS very_difficult;
- IF sensors_bandwidth IS very_low THEN Experiment IS very_difficult;
- IF sensors_bandwidth IS high THEN Experiment IS convenient;
- IF sensors_bandwidth IS very_high THEN Experiment IS very_convenient.

The last developed subsystem labeled as Problem is composed of a single Mamdani-type fuzzy logic system with three inputs: problem_labels, problem_type, and problem_component. Each input contains the Gaussian membership functions with numbers corresponding to the answers presented in Appendix A. The output problem contains five Gaussian membership functions (novelty, semi_supervised, multiclass, regression, and everything) and output problem_monitored contains three Gaussian membership functions (lower_freq_comp, wide_freq_range, and higher_freq_comp) that evenly cover the value range from 0 to 1. The list below contains system rules:

- IF problem_labels IS None THEN Problem IS novelty;
- IF problem_labels IS Few_examples AND problem_type IS Fault_detection THEN Problem IS novelty;
- IF problem_labels IS Few_examples AND problem_type IS NOT Fault_detection THEN Problem IS semi_supervised;
- IF problem_labels IS Multiple_examples AND problem_type IS Fault_detection THEN Problem IS novelty;
- IF problem_labels IS Multiple_examples AND problem_type IS Assessment _of_the_condition THEN Problem IS semi_supervised;
- IF problem_labels IS Multiple_examples AND problem_type IS Assessment _of_the_temporal_condition THEN Problem IS regression;
- IF problem_labels IS Multiple_examples AND problem_type IS Multiple_problems THEN Problem IS everything;
- IF problem_component IS Shaft THEN Problem_monitored IS lower_freq_comp;
- IF problem_component IS Multiple_components THEN Problem_monitored IS wide_freq_range;
- IF problem_component IS Other THEN Problem_monitored IS wide_freq_range;
- IF problem_component IS Bearing THEN Problem_monitored IS higher_freq_comp;
- IF problem_component IS Gear THEN Problem_monitored IS higher_freq_comp.

## Appendix C. Developed Rules for Signal Processing Methods Filter

This appendix presents the developed rules. The rules were developed based on knowledge of fault detection in rotating machinery. A shortlist of linguistic rules is given below:

- If a phase marker signal is acquired, the time-synchronous signal averaging should be considered.
- Shaft-related faults are detected by analyzing the speed signal.
- Many faults manifest their presence as additional bands visible in the signal spectrum or its envelope.
- From the phase marker signal, one can obtain an instantaneous velocity value.
- It is useful to remove the constant value before processing the signals.

These and similar findings were used to develop the rules. Due to their number, only those concerning the significant processing methods are presented:

1. Linear detrending (detrend_lin)
  - IF Variability IS very_low THEN detrend_lin IS high;
  - IF Variability IS low THEN detrend_lin IS high;
  - IF Variability IS medium THEN detrend_lin IS high;
  - IF Variability IS high THEN detrend_lin IS high;
  - IF Variability IS very_high THEN detrend_lin IS high.
2. Signal envelope (Envelope)
  - IF Variability IS very_low AND problem_component IS NOT Shaft THEN Envelope IS very_high;
  - IF Variability IS low AND problem_component IS NOT Shaft THEN Envelope IS very_high;
  - IF Variability IS medium AND problem_component IS NOT Shaft THEN Envelope IS very_high;
  - IF Variability IS high AND problem_component IS NOT Shaft THEN Envelope IS high;
  - IF Variability IS very_high AND problem_component IS NOT Shaft THEN Envelope IS high;
  - IF problem_component IS Shaft THEN Envelope_TD IS medium.
3. Frequency demodulation (Frequency_modulation)
  - IF phase_marker IS Yes THEN Frequency_modulation IS very_high;
  - IF phase_marker IS No AND Variability IS very_low THEN Frequency_modulation IS high;
  - IF phase_marker IS No AND Variability IS low THEN Frequency_modulation IS high;
  - IF phase_marker IS No AND Variability IS medium THEN Frequency_modulation IS high;
  - IF phase_marker IS No AND Variability IS high THEN Frequency_modulation IS medium;
  - IF phase_marker IS No AND Variability IS very_high THEN Frequency_modulation IS medium.
4. Extraction of instantaneous frequency (Instantaneous_frequency)
  - IF Variability IS very_low THEN Instantaneous_frequency IS low;
  - IF Variability IS low THEN Instantaneous_frequency IS medium;
  - IF Variability IS medium THEN Instantaneous_frequency IS medium;
  - IF Variability IS high THEN Instantaneous_frequency IS high;
  - IF Variability IS very_high THEN Instantaneous_frequency IS high.
5. Extraction of instantaneous frequency from phase marker (Instantaneous_frequency_from_KF)
  - IF phase_marker IS No THEN Instantaneous_frequency_from_KF IS very_low;
  - IF phase_marker IS Yes AND Variability IS very_low THEN Instantaneous _frequency_from_KF IS very_high;
  - IF phase_marker IS Yes AND Variability IS low THEN Instantaneous _frequency_from_KF IS very_high;
  - IF phase_marker IS Yes AND Variability IS medium THEN Instantaneous _frequency_from_KF IS high;
  - IF phase_marker IS Yes AND Variability IS high THEN Instantaneous _frequency_from_KF IS medium;
  - IF phase_marker IS Yes AND Variability IS very_high THEN Instantaneous _frequency_from_KF IS low.
6. Signal integration (integration)
  - IF sensors_type IS Accelerometer AND problem_component IS Shaft THEN integration IS very_high;
  - IF sensors_type IS Accelerometer AND problem_component IS Multiple_components THEN integration IS very_high;
  - IF sensors_type IS Accelerometer AND problem_component IS Other THEN integration IS medium;
  - IF sensors_type IS Accelerometer AND problem_component IS Gear THEN integration IS low;
  - IF sensors_type IS Vibrometer THEN integration IS low.
7. Processing time signal into order domain (Order_spectrum)
  - IF phase_marker IS Yes THEN Order_spectrum IS very_high;
  - IF phase_marker IS No THEN Order_spectrum IS very_low.
8. Signal frequency spectrum (Spectrum_FD)
  - IF phase_marker IS Yes THEN Spectrum_FD IS very_high;
  - IF phase_marker IS No AND Variability IS very_low THEN Spectrum_FD IS very_high;
  - IF phase_markern IS No AND Variability IS low THEN Spectrum_FD IS very_high;
  - IF phase_marker IS No AND Variability IS medium THEN Spectrum_FD IS high;
  - IF phase_marker IS No AND Variability IS high THEN Spectrum_FD IS medium;
  - IF phase_marker IS No AND Variability IS very_high THEN Spectrum_FD IS medium.
9. Time synchronous averaging (time_synch_averaging)
  - IF phase_marker IS Yes AND problem_component IS Gear THEN time_synch_averaging IS very_high;
  - IF phase_marker IS Yes AND problem_component IS Bearing THEN time_synch_averaging IS very_high;
  - IF phase_marker IS Yes AND problem_component IS Multiple_components THEN time_synch_averaging IS very_high;
  - IF phase_marker IS Yes AND problem_component IS Other THEN time_synch_averaging IS high;
  - IF phase_marker IS Yes AND problem_component IS Shaft THEN time_synch_averaging IS low;
  - IF phase_marker IS No THEN time_synch_averaging IS very_low.

## Appendix D. Developed Rules for the Model Selector

The rules for the Model group subsystem were developed based on experience related to training different types of machine learning models. Neural networks provide good results for a large number of data samples. This is especially true for high-dimensional data where deep models are favored. On the other hand, algorithms based on distance or decision trees perform better for data with a low number of features. Algorithms based on clustering are recommended in other cases. Based on these empirical formulations, fuzzy rules were developed and presented in the following list:

1. Rules for trees output
  - IF sample_number IS very_low AND features_number IS very_low THEN trees IS very_high;
  - IF sample_number IS low AND features_number IS very_low THEN trees IS very_high;
  - IF sample_number IS medium AND features_number IS very_low THEN trees IS very_high;
  - IF sample_number IS very_low AND features_number IS low THEN trees IS very_high;
  - IF sample_number IS low AND features_number IS low THEN trees IS very_high;
  - IF sample_number IS medium AND features_number IS low THEN trees IS very_high;
  - IF sample_number IS very_low AND features_number IS medium THEN trees IS high;
  - IF sample_number IS low AND features_number IS medium THEN trees IS high;
  - IF sample_number IS medium AND features_number IS medium THEN trees IS very_high;
  - IF features_number IS high THEN trees IS medium;
  - IF features_number IS very_high THEN trees IS low.
2. Rules for distance output
  - IF sample_number IS NOT very_high AND features_number IS very_low THEN distance IS very_high;
  - IF sample_number IS NOT very_high AND features_number IS low THEN distance IS very_high;
  - IF sample_number IS NOT very_high AND features_number IS medium THEN distance IS high;
  - IF sample_number IS very_high AND features_number IS very_low THEN distance IS high;
  - IF sample_number IS very_high AND features_number IS low THEN distance IS medium;
  - IF sample_number IS very_high AND features_number IS medium THEN distance IS low;
  - IF features_number IS high THEN distance IS very_low;
  - IF features_number IS very_high THEN distance IS very_low.
3. Rules for clustering output
  - IF sample_number IS very_low THEN clustering THEN medium;
  - IF sample_number IS low AND features_number IS low THEN clustering IS high;
  - IF sample_number IS low AND features_number IS very_low THEN clustering IS very_high;
  - IF sample_number IS medium AND features_number IS very_low THEN clustering IS very_high;
  - IF sample_number IS high AND features_number IS very_low THEN clustering IS very_high;
  - IF sample_number IS medium AND features_number IS low THEN clustering IS very_high;
  - IF sample_number IS high AND features_number IS low THEN clustering IS very_high;
  - IF sample_number IS medium AND features_number IS medium THEN clustering IS very_high;
  - IF sample_number IS high AND features_number IS medium THEN clustering IS very_high;
  - IF sample_number IS very_high AND features_number IS high THEN clustering IS medium;
  - IF features_number IS high THEN clustering IS medium;
  - IF features_number IS very_high THEN clustering IS very_low.
4. Rules for networks output
  - IF sample_number IS very_low THEN networks IS low;
  - IF sample_number IS low AND features_number IS NOT very_low THEN networks IS medium;
  - IF sample_number IS low AND features_number IS very_low THEN networks IS high;
  - IF sample_number IS medium AND features_number IS medium THEN networks IS high;
  - IF sample_number IS medium AND features_number IS low THEN networks IS very_high;
  - IF sample_number IS medium AND features_number IS very_low THEN networks IS very_high;
  - IF sample_number IS high AND features_number IS medium THEN networks IS very_high;
  - IF sample_number IS high AND features_number IS low THEN networks IS very_high;
  - IF sample_number IS high AND features_number IS very_low THEN networks IS very_high;
  - IF sample_number IS high AND features_number IS high THEN networks IS high;
  - IF sample_number IS very_high AND features_number IS medium THEN networks IS very_high;
  - IF sample_number IS very_high AND features_number IS low THEN networks IS very_high;
  - IF sample_number IS very_high AND features_number IS very_low THEN networks IS very_high;
  - IF sample_number IS very_high AND features_number IS high THEN networks IS high;
  - IF features_number IS very_high THEN networks IS medium.
5. Rules for deep output
  - IF sample_number IS very_low THEN deep IS very_low;
  - IF sample_number IS low THEN deep IS low;
  - IF sample_number IS medium THEN deep IS medium;
  - IF sample_number IS high AND features_number IS medium THEN deep IS high;
  - IF sample_number IS high AND features_number IS high THEN deep IS very_high;
  - IF sample_number IS high AND features_number IS very_high THEN deep IS very_high;
  - IF sample_number IS high AND features_number IS medium THEN deep IS very_high;
  - IF sample_number IS very_high AND features_number IS high THEN deep IS very_high;
  - IF sample_number IS very_high AND features_number IS very_high THEN deep IS very_high.

The rules developed for the problem class subsystem were also derived from the experience in the development of machine learning models. If the number of classes is up to two or the number of unique classes is greater, but only one target class has good representation, then one should consider the novelty detection approach. In other cases, the training of the classifier is a good solution. Based on these statements, rules were developed and written in the form of the following list:

1. Rules for novelty_detection (nd) output
  - IF targets_number IS low THEN novelty_detection IS high;
  - IF targets_number IS medium AND targets_samples_std IS NOT low THEN novelty_detection IS medium;
  - IF targets_number IS high THEN novelty_detection IS low.
2. Rules for classification (class) output
  - IF targets_number IS NOT low AND targets_samples_std IS high THEN classification IS medium;
  - IF targets_number IS NOT low AND targets_samples_std IS NOT high THEN classification IS high;
  - IF targets_number IS low AND targets_samples_std IS NOT high THEN classification IS medium;
  - IF targets_number IS low AND targets_samples_std IS high THEN classification IS low.

The rules for the *i*-th model type were developed by training different models based on the prepared sets to be trained. Measures for these sets and the effectiveness of the developed models were calculated. The rules were developed, and their list is presented below:

1. Rules developed for decision tree
  - IF trees IS very_high AND classification IS high THEN decision_tree IS very_high;
  - IF trees IS very_high AND classification IS medium THEN decision_tree IS high;
  - IF trees IS high AND classification IS high THEN decision_tree IS high;
  - IF trees IS high AND classification IS medium THEN decision_tree IS medium;
  - IF trees IS medium AND classification IS NOT low THEN decision_tree IS medium;
  - IF trees IS low AND classification IS NOT low THEN decision_tree IS low;
  - IF trees IS very_low AND classification IS NOT low THEN decision_tree IS very_low;
  - IF classification IS low THEN decision_tree IS low.
2. Rules developed for k-means classifier (KMC)
  - IF clustering IS very_high AND classification IS high AND clusters_to_targets_ratio IS NOT below_identity AND clusters_mean_diversity IS low THEN kmc IS very_high;
  - IF clustering IS very_high AND classification IS medium AND clusters_to_targets_ratio IS NOT below_identity AND clusters_mean_diversity IS low THEN kmc IS high;
  - IF clustering IS very_high AND classification IS high AND clusters_to_targets_ratio IS below_identity AND clusters_mean_diversity IS low THEN kmc IS high;
  - IF clustering IS very_high AND classification IS medium AND clusters_to_targets_ratio IS below_identity AND clusters_mean_diversity IS low THEN kmc IS medium;
  - IF clustering IS very_high AND classification IS NOT low AND clusters_mean_diversity IS NOT low THEN kmc IS medium;
  - IF clustering IS high AND classification IS high AND clusters_mean_diversity IS NOT high THEN kmc IS high;
  - IF clustering IS high AND classification IS high AND clusters_mean_diversity IS high THEN kmc IS medium;
  - IF clustering IS high AND classification IS medium THEN kmc IS medium;
  - IF clustering IS medium AND classification IS NOT low THEN kmc IS medium;
  - IF clustering IS low AND classification IS NOT low THEN kmc IS low;
  - IF clustering IS very_low AND classification IS NOT low THEN kmc IS very_low;
  - IF classification IS low THEN kmc IS low.
3. Rules developed for k-nearest neighbor classifier (kNNC)
  - IF distance IS very_high AND classification IS high AND clusters_mean_diversity IS NOT high THEN knnc IS very_high;
  - IF distance IS very_high AND classification IS medium AND clusters_mean_diversity IS NOT high THEN knnc IS high;
  - IF distance IS very_high AND classification IS high AND clusters_mean_diversity IS high THEN knnc IS high;
  - IF distance IS very_high AND classification IS medium AND clusters_mean_diversity IS high THEN knnc IS medium;
  - IF distance IS high AND classification IS high AND clusters_mean_diversity IS NOT high THEN knnc IS high;
  - IF distance IS high AND classification IS medium AND clusters_mean_diversity IS NOT high THEN knnc IS medium;
  - IF distance IS high AND classification IS NOT low AND clusters_mean_diversity IS high THEN knnc IS medium;
  - IF distance IS medium AND classification IS NOT low THEN knnc IS medium;
  - IF distance IS low AND classification IS NOT low THEN knnc IS low;
  - IF distance IS very_low AND classification IS NOT low THEN knnc IS very_low;
  - IF classification IS low THEN knnc IS low.
4. Rules developed for multilayer perceptron (MLP)
  - IF networks IS very_high AND classification IS high AND clusters_to_targets_ratio IS NOT below_identity AND clusters
    -_mean_diversity IS NOT high THEN mlp IS very_high;
  - IF networks IS very_high AND classification IS medium AND clusters_to_targets_ratio IS NOT below_identity AND clusters_mean_diversity IS NOT high THEN mlp IS high;
  - IF networks IS very_high AND classification IS NOT low AND clusters_to_targets_ratio IS below_identity AND clusters_mean_diversity IS NOT high THEN mlp IS high;
  - IF networks IS very_high AND classification IS NOT low AND clusters_mean_diversity IS high THEN mlp IS high;
  - IF networks IS high AND classification IS high AND clusters_mean_diversity IS NOT high THEN mlp IS high;
  - IF networks IS high AND classification IS medium AND clusters_mean_diversity IS NOT high THEN mlp IS medium;
  - IF networks IS high AND classification IS NOT low AND clusters_mean_diversity IS high THEN mlp IS medium;
  - IF networks IS medium AND classification IS NOT low THEN mlp IS medium;
  - IF networks IS low AND classification IS NOT low THEN mlp IS low;
  - IF networks IS very_low AND classification IS NOT low THEN mlp IS very_low;
  - IF classification IS low THEN mlp IS low.
5. Rules developed for random forest
  - IF trees IS very_high AND classification IS high THEN random_forest IS very_high;
  - IF trees IS very_high AND classification IS medium THEN random_forest IS high;
  - IF trees IS high AND classification IS high THEN random_forest IS high;
  - IF trees IS high AND classification IS medium THEN random_forest IS medium;
  - IF trees IS medium AND classification IS NOT low THEN random_forest IS medium;
  - IF trees IS low AND classification IS NOT low THEN random_forest IS low;
  - IF trees IS very_low AND classification IS NOT low THEN random_forest IS very_low;
  - IF classification IS low THEN random_forest IS low.
